# Comparison of Direct Fluorescence Assay and Real-Time RT-PCR as Diagnostics for Respiratory Syncytial Virus in Young Children

**DOI:** 10.1155/2011/781919

**Published:** 2011-12-14

**Authors:** Caroline F. Shafik, Emad W. Mohareb, Fouad G. Youssef

**Affiliations:** Viral and Zoonotic Diseases Research Program, U.S. Naval Medical Research Unit No. 3, Cairo 11517, Egypt

## Abstract

Respiratory syncytial virus (RSV) is the major cause of lower respiratory tract infections in children worldwide. Early detection of RSV is critical to initiate proper care. Two methods, the direct fluorescence assay (DFA) and the real-time reverse-transcription polymerase chain reaction (rt-RT-PCR), that are used for RSV detection were compared. A total of 451 nasopharyngeal aspirates from children 5 years of age or less were tested for RSV using both methods. The overall prevalence rate of the RSV among the children was 23.7% with a significantly higher prevalence among children under the age of 6 months of age when compared to other age groups. The sensitivity of DFA in comparison to rt-RT-PCR was highest (86%) during the first 3 days of symptoms onset and decreased gradually till it reached 65% after the first week. The specificity of DFA in comparison to rt-RT-PCR ranged between 99 and 100% irrespective of the date of collection. We concluded that, although the rt-RT-PCR is more sensitive for RSV detection, the DFA offers a reliable point-of-care alternative detection method especially during the first few days of illness.

## 1. Introduction

Respiratory syncytial virus (RSV) is a negative sense single-stranded RNA virus belonging to *Pneumovirus* genus, subfamily *Pneumovirinae*. RSV is considered a major cause of severe lower respiratory tract infections in children less than two years of age worldwide [1]. Morbidity and mortality are greatly increased in children with bacterial coinfections or superinfections [2]. Therefore, early detection of the virus is a critical step in the initiation of proper care, and the prevention of further spread of the virus in public places such as schools and health care facilities. Direct Fluorescence assay (DFA) is a conventional method that is frequently used in the clinical setting for the detection of respiratory viruses including RSV. However, nucleic acid detection methods have proven to be more sensitive for RSV detection [3]. Some countries, particularly developing countries, cannot afford to use nucleic acid detection methods within hospital laboratories due to high cost and lack of technical expertise. In this study, we compared the sensitivity and specificity of the DFA against real-time reverse transcriptase polymerase chain reaction (rt-RT-PCR) as a point-of-care method for RSV detection. Comparison was also made between both assays in relation to the days after onset of symptoms.

## 2. Materials and Methods

Four hundred and fifty one specimens were collected between December, 2006 and November, 2007 from patients presenting to Abou El Rish Pediatric Hospital, Cairo, Egypt, with acute lower respiratory tract infection (ALRTI) as per the WHO case definition (WHO/CDS/2004.25). For each patient that met the criteria, a nasopharyngeal aspirate (NPA) was collected using the infant mucous extractor (ARGYLE DeLee, Kendall, USA). Clinical history including the date of onset of symptoms was recorded. Virus transport media was added to each NPA and was split into two aliquots, one for direct fluorescent assay (DFA) and the second for nucleic acid extraction. For DFA, an epithelial cell suspension in phosphate buffered saline was prepared for each sample and air-dried on multiwell Teflon slides. The Respiratory Panel 1 DFA kit (LIGHT DIAGNOSTICS Millipore, Calif, USA) was used for DFA. Total nucleic extraction was performed using MagMax Express96 (Applied Biosystems) and the Total Nucleic Acid Isolation Kit (Ambion) per manufacturer's instructions. rt-RT-PCR was performed as per Centers for Disease Control and Prevention (CDC), Atlanta, Ga, USA using CDC's primers. A sample was considered positive for RSV if its cycle threshold (Ct) was ≤38.

Statistical significance was determined by Chi-square and Fisher's exact *t*-test using EpiInfo software, version 6.04

In this study, rt-RT-PCR served as the standard test, and sensitivity and specificity of DFA were calculated on the basis the PCR results. Sensitivity of DFA was calculated by dividing the number of samples positive for RSV by both DFA and rt-RT-PCR by the total number of samples positive by rt-RT-PCR. Specificity of DFA was calculated by dividing the number of samples negative by both DFA and rt-RT-PCR by the total number of samples negative by rt-RT-PCR.

Positive Predictive Value (PPV) of DFA was calculated by dividing the number of samples positive by both DFA and rt-RT-PCR by the total number of DFA positive samples. negative predictive value (NPV) was calculated by dividing the number of samples negative by both DFA and rt-RT-PCR by the total number of DFA negative samples.

## 3. Results

**Figure 1:**
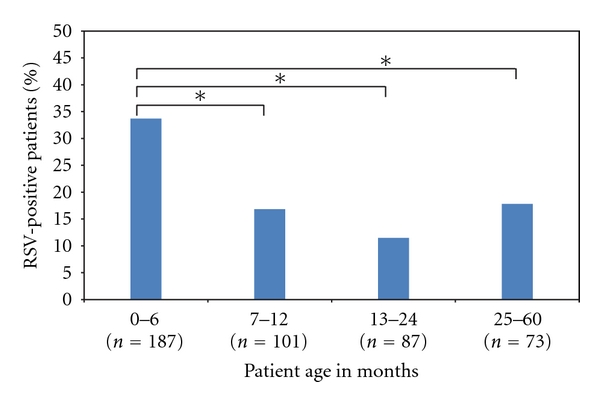
Age distribution of the RSV-positive patients. *Statistically significant difference (*P* < 0.05).

A total of 451 patients were enrolled in this study. The age range of patients was 0–60 months, 41.5% were between 0 and 6 months of age. The prevalence rate of RSV in this study population by the two assays was 23.7% (107/451). Patients aged 0–6 months had a statistically significant higher rate of RSV infection compared to in the other age groups (Figure 1).

The overall sensitivity of DFA for RSV detection was 77.8% and specificity 99.6%. The PPV for DFA was 98.6%, and the NPV was 94%.

**Figure 2:**
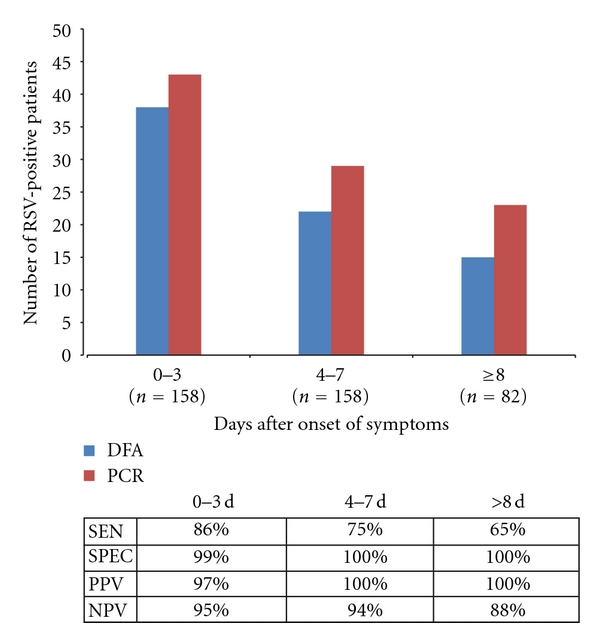
RSV-positive samples detected by each method at different times after onset of symptoms. SEN: sensitivity of DFA, SPEC: specificity of DFA, PPV: positive predictive value, and NPV: negative predictive value.

We examined the effect of sample collection date in relation to date of onset of symptoms on the DFA sensitivity for detection of RSV. The DFA was 86% sensitive and 99% specific between 0 and 3 days after onset of symptoms. The sensitivity of DFA dropped to 75.8% when samples were collected 4–7 days after onset of symptoms and was 100% specific. DFA was only 65% sensitive when samples were collected 8 days and more after symptom onset, and the specificity remained at 100%, indicating that the assay is still reliable (Figure 2).

## 4. Conclusion

The rate of RSV prevalence was successfully monitored using two clinical diagnostic assays. The rate of RSV infection was significantly higher among children aged 0–6 months old, which is in accordance with other studies [4]. Both assays are very specific and have good predictive value for diagnosis. In general, rt-RT-PCR is more sensitive than DFA for detection of RSV. The downfall of this technique is the expense and that it is not currently available in many clinical settings, especially in developing countries. Therefore, we also examined the commonly used DFA assay for the detection of RSV in a clinical setting. DFA still offers a reliable option for the detection of RSV especially for patients tested with the first 3 days after onset of symptoms. Successful RSV testing during the first days of illness enables the physician to make the best decision regarding treatment for the child and prevent the spread of infection especially within health care facilities.
